# The Transcriptome of Type I Murine Astrocytes under Interferon-Gamma Exposure and Remyelination Stimulus

**DOI:** 10.3390/molecules22050808

**Published:** 2017-05-15

**Authors:** Anna Kudriaeva, Vladimir V. Galatenko, Diana V. Maltseva, Nadezhda A. Khaustova, Ekaterina Kuzina, Alexander G. Tonevitsky, Alexander Gabibov, Alexey Belogurov

**Affiliations:** 1 Shemyakin-Ovchinnikov Institute of Bioorganic Chemistry, Russian Academy of Sciences, 117997 Moscow, Russia; anna.kudriaeva@gmail.com (A.K.); ekaterina.kuzina@yale.edu (E.K.); gabibov@mx.ibch.ru (A.G.); 2 Department of Mathematical Analysis, Faculty of Mechanics and Mathematics, Lomonosov Moscow State University, 119991 Moscow, Russia; vgalat@msu.ru; 3 Big Data and Information Retrieval School, Faculty of Computer Science, National Research University Higher School of Economics, 125319 Moscow, Russia; 4 SRC Bioclinicum, 115088 Moscow, Russia; d.maltseva@bioclinicum.com (D.V.M.); khaunadia@gmail.com (N.A.K.); 5 P. Hertsen Moscow Oncology Research Institute, 125284 Moscow, Russia; tonevitsky@mail.ru; 6 Institute of Fundamental Medicine and Biology, Kazan Federal University, 420008 Kazan, Russia

**Keywords:** astrocytes, transcriptome, Affymetrix, interferon-gamma, benztropine, immunoproteasome, PA28/11S/REG, antigen presentation, major histocompatibility complex, microRNA

## Abstract

Astrocytes are considered to be an important contributor to central nervous system (CNS) disorders, particularly multiple sclerosis. The transcriptome of these cells is greatly affected by cytokines released by lymphocytes, penetrating the blood–brain barrier—in particular, the classical pro-inflammatory cytokine interferon-gamma (IFNγ). We report here the transcriptomal profiling of astrocytes treated using IFNγ and benztropine, a putative remyelinization agent. Our findings indicate that the expression of genes involved in antigen processing and presentation in astrocytes are significantly upregulated upon IFNγ exposure, emphasizing the critical role of this cytokine in the redirection of immune response towards self-antigens. Data reported herein support previous observations that the IFNγ-induced JAK-STAT signaling pathway may be regarded as a valuable target for pharmaceutical interventions.

## 1. Introduction

Autoimmune neurodegeneration—also known as multiple sclerosis (MS)—is caused by adaptive immunity that recognizes self-antigens forming the myelin sheath, covering the axons of neurons. Oligodendrocytes are specialized cells expressing myelin proteins, and are the main target of the immune system during MS triggering and development [1]. Astrocytes are star-shaped glial cells in the brain and spinal cord, and significantly tune the autoreactive cellular response, releasing potentially neurotoxic molecules, including inflammatory cytokines, glutamate, nitric oxide, and reactive oxygen species [2]. One of the most important hallmarks of MS is the induction of inflammation in the central nervous system (CNS), orchestrated by lymphocytes infiltrating via the blood–brain barrier (BBB). In turn, inflammation is driven by secreted cytokines, of which interferon-gamma (IFNγ) is thought to be one of the most important [3]. Recent data also indicate that the muscarinic acetylcholine receptor antagonist benztropine—an approved drug for Parkinson’s disease—may be considered as a potential agent for MS treatment [4].

Current progress in the evaluation of brain-related transcriptomes of tissue samples and cell populations [5] is consistently complemented with mass-spectromic profiling of resident brain cells [6,7] and single-cell RNA sequencing [8,9]. To date, the Glia Open Access Database (GOAD) contains several tens of mRNA expression profiles for glia cells [10]. The abundance of mRNA in myelin was reported in Reference [11]. Zhang et al. generated a transcriptome database for neurons, astrocytes, oligodendrocyte precursor cells, newly-formed oligodendrocytes, myelinating oligodendrocytes, microglia, endothelial cells, and pericytes from mouse cerebral cortex using RNA sequencing [12]. As an another example, high-throughput RNA sequencing of brain cells was used to analyze the consequences of the loss of a key transcription factor, zinc finger protein 191 [13]. The effect of benztropine on α-synuclein-overexpressing primary rat oligodendrocyte progenitor cells, utilizing transcriptome sequencing, was evaluated in Reference [14]. Recently, we reported transcriptome profiling of a primary culture of oligodendrocytes under inflammation conditions and remyelination stimulus, suggesting significant elevation of mRNA encoding catalytic subunits of immunoproteasome and major histocompatibility complex (MHC) class I molecules, which means increased capabilities of antigen presentation by IFNγ-treated oligodendrocytes [15]. In the present study, we aimed at analyzing the transcriptome of murine astrocytes in the background state, and to compare it with those under exposure to IFNγ, benztropine, or a combination of the two.

## 2. Results and Discussion

### 2.1. Analysis of Astrocyte Transcriptional Markers

**Figure 1:**
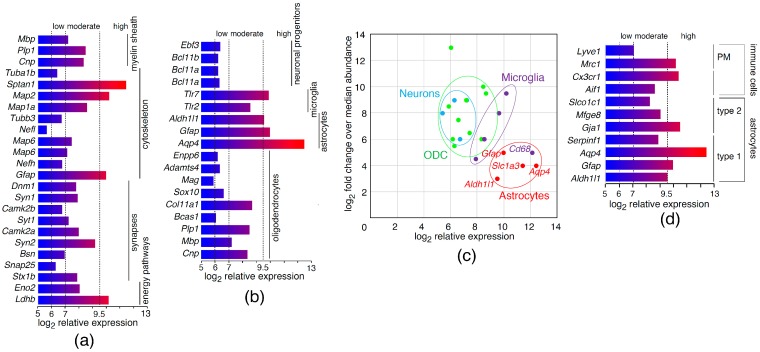
Abundant and enriched transcripts in murine cerebral cells. Expression profile of mRNA from cultured astrocytes coding for proteins enriched in (**a**) brain cells and (**b**) different cerebral cell types according to Sharma et al. [6]; (**c**) Scatter plot of log_2_ fold expression versus log_2_ fold change over median abundance in different brain cell types, according to Sharma et al. [6]; (**d**) Typing of cultured astrocytes in accordance with markers reported in Zeisel et al. [8].

We obtained a primary culture of cerebral cells which were significantly enriched with murine brain astrocytes. Cerebral cells are known to have distinct transcriptional and proteomic profiles. Studies of murine brain, utilizing mass-spectrometry, revealed the top 40 most abundant and enriched proteins [6] of the myelin sheath (*Mbp*, *Plp1*, and *Cnp*), the cytoskeleton (*Tuba1b*, *Actb*, *Sptan1*, *Map2*, *Map1a*, *Tubb3*, *Nefl*, *Map6*, *Nefh*, and *Gfap*), synapses (*Dnm1*, *Syn1*, *Camk2b*, *Syt1*, *Camk2a*, *Syn2*, *Bsn*, *Snap25*, and *Stx1b*), and glycolysis, as well as energy pathways (*Aldoa*, *Eno2*, and *Ldhb*). According to our data, mRNA related to these proteins was highly abundant in the primary culture of astrocytes obtained from mouse brain, except for *Snap25*, *Camk2b*, *Nefl*, and *Tuba1b* (Figure 1a). In order to more precisely characterize the types of cultured cells, we analyzed the levels of mRNA coding for the cell-type-specific markers. The mRNA encoding the proteins related to astrocytes (*Aqp4*, *Gfap*, and *Aldh1l1*) and microglia (*Iba1*, *Tlr2*, and *Tlr7*) [6] were significantly upregulated (Figure 1b). Importantly, we found evident correlation between the levels of proteins that are specific for astrocytes (as reported by Sharma et al.), and the levels of respective mRNA, indicating that enhanced levels of these transcripts have distinct physiological meanings. The plotting of overexpressed proteins in different cell types, according to Reference [6], against expression profiles of cultured cerebral cells confirmed the predominance of astrocytes and the existence of minor populations of CD68-positive cells, most likely representing macrophages (Figure 1c). In their study, Zeisel et al. [8] showed that astrocytes form two subclasses, distinguished by differential expression of *Gfap* (type I) and *Mfge8* (type II), and the existence of two types of immune cells, which are characterized by increased expression of *Aif1* and *Cx3cr1*: the tissue-resident macrophages of the brain (microglia) and perivascular macrophages (PM), expressing *Mrc1* and *Lyve1*. Our data suggest that the cultured astrocytes were type I, while the high level of mRNA coding for *Mrc1* suggests the presence of perivascular macrophages (Figure 1d).

### 2.2. Transcriptome Profiling of Astrocytes upon IFNγ and Benztropine Exposure

**Figure 2:**
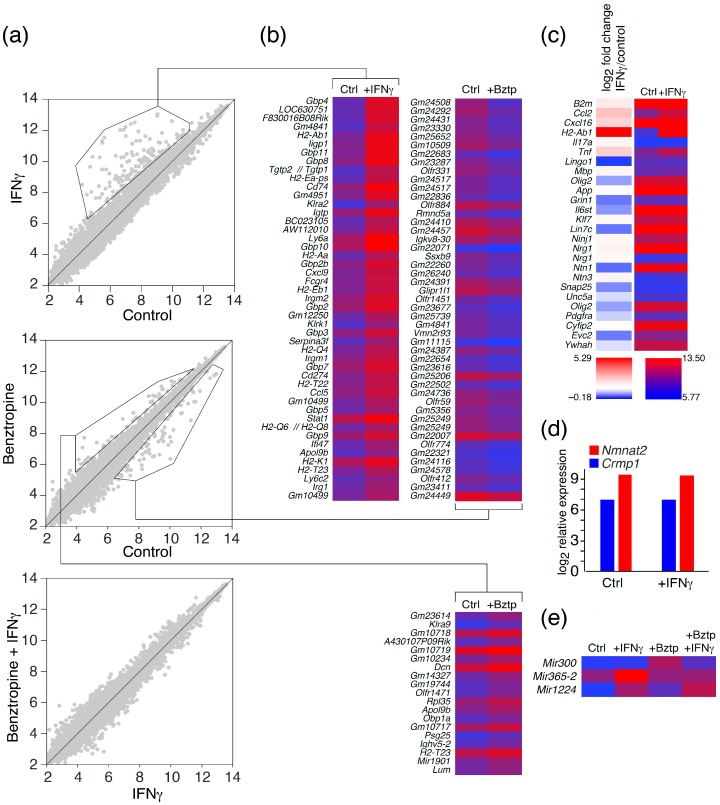
Differentially-expressed genes in a culture of astrocytes in response to inflammation and remyelination stimulus. (**a**,**b**) Expression profile of differentially-expressed genes in control astrocytes (Control) and astrocytes exposed to interferon-gamma (IFNγ), benztropine (Bztp), or both agents simultaneously; (**c**) Differential expression of genes reported in Evangelidou et al. [16] in a culture of astrocytes subjected to IFNγ in comparison with the control; (**d**) Expression of *Nmnat2* and *Crmp1* genes in a culture of astrocytes under IFNγ exposure in comparison with the control; (**e**) Expression of microRNAs (miR) in astrocyte culture, treated with benztropine, IFNγ, or their combination, as compared to the control.

Transcriptome profiling revealed 474 and 150 genes that were upregulated and downregulated, respectively, in a primary culture of murine cerebral cells, significantly enriched with astrocytes, upon IFNγ administration; exposure to benztropine increased the expression of 131 genes and downregulated the expression of 125 genes (Figure 2a,b). The transcriptomes of astrocytes subjected to IFNγ and benztropine simultaneously did not differ from those exposed to only IFNγ. Among pathways that were affected by IFNγ may be listed immune and defense responses, antigen processing and presentation, response to viruses and interferon-inducible GTPases; benztropine mostly affected receptor pathways, including rhodopsin-like G protein-coupled receptors and olfactory receptors (please refer to the Supplementary data). Utilizing quantitative RT-PCR, Evangelidou and colleagues [16] measured transcripts in mouse spinal cord during induction of experimental autoimmune encephalomyelitis (EAE). The authors showed that a number of genes involved in inflammation and immune responses, specifically, *B2m*, *Ccl2*, *Cxcl16*, *H2-Ab1*, *Il6*, *Il17a*, and *Tnf* were upregulated, while *Lingo1*, *Mbp*, *Olig2*, *App*, *Grin1*, *Il6st*, *Klf7*, *Lin7c*, *Ninj1*, *Nrg1*, *Ntn1*, *Ntn3*, *Ptma1*, *Snap25*, *Unc5a*, *Bmi1*, *Olig2*, *Pdgfra*, *Cyfip2*, *Evc2*, *Tmem1*, and *Ywhah* were downregulated. Our data strongly correlate with this report (Figure 2c), indicating significantly increased expression of MHC class II molecules. In our work, we failed to detect any increase in the levels of mRNAs encoding *Crmp1* or *Nmnat2* (Figure 2d), which were increased in response to EAE, as shown by Solga et al. using Illumina-based RNA sequencing of glia from Iba1-EGFP C57BL/6 mice [17]. The effect of benztropine on astrocytes was significantly less pronounced. Administration of benztropine enhanced the level of mRNA encoding killer cell lectin-like receptor subfamily A member 9 (*Klra*9), decorin (*Dcn*), neutrophil gelatinase-associated lipocalin (*Lcn2*), involved in innate immunity, and the pheromone transporter major urinary protein 4 (*Mup4*). On the other hand, the level of mRNA encoding protein RMD5 homolog A (*Rmnd5a*, involved in proteasome-mediated ubiquitin-dependent protein catabolic processes) and hepcidin liver-produced hormone (*Hamp*, the main circulating regulator of iron absorption and distribution across tissues) were downregulated under benztropine exposure. Interestingly, benztropine—like IFNγ—upregulated the *H2-T3* gene coding for H-2 class I histocompatibility antigen, TLA(B) alpha chain.

We utilized Affymetrix microarray, which contained probesets for the protein-coding genes and for more than 600 microRNAs (miRs). Analysis of differentially-expressed miRs (Figure 2e) revealed that benztropine enhanced the expression of miR-300 (which is upregulated in glioma tissues), and suppressed the differentiation of glioma stem-like cells to astrocytes by targeting LZTS2 [18]. IFNγ increased the expression of miR-365, which, on the other hand, inhibits the proliferation of malignant melanoma by targeting NRP1 [19], and miR-1224, which inhibits tumor-associated activity in malignant gliomas by targeting CREB1 [20]. Nonetheless, analyses of the amounts of mRNAs encoding NRP1, CREB1, and LZTS2 did not reveal any differences in level upon IFNγ and benztropin treatment. We therefore suggest that the observed changes in miR levels are not physiologically relevant.

### 2.3. Changes in Transcription of Genes Related to Antigen Proteolysis and Presentation in Astrocytes in Response to IFNγ

**Figure 3:**
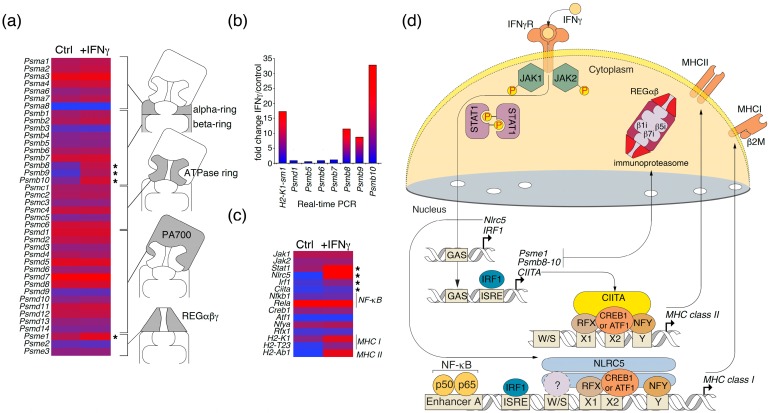
Expression profile of differentially-expressed genes related to (**a**) proteasomes and (**c**) JAK-STAT signaling in control astrocytes (Ctrl) and in astrocytes under IFNγ stimuli; (**b**) Analysis of the level of transcription of immunoproteasome-related genes using real-time PCR. Significantly upregulated genes are marked by an asterisk; (**d**) IFNγ-regulated transcription of immunoproteasome-related genes and genes coding for MHC class I/II molecules through JAK-STAT signaling.

Recently, we showed that the immunoproteasome has a distinct pathogenic role in the development of autoimmune neurodegeneration by targeting cytotoxic lymphocytes against oligodendrocytes [21] through ubiquitin-independent hydrolysis of myelin basic protein (MBP) [22]. Proteasome-mediated MBP hydrolysis was interrupted by glatiramer acetate [23], and was significantly enhanced in cells upon IFNγ exposure [24]. The proteasome is a major supplier of MHC class I-associated peptides; however, it may also be involved in the generation of MHC class II-restricted epitopes [25]. Therefore, we analyzed changes in the expression of mRNA encoding proteasome-related proteins in a culture of astrocytes (Figure 3a). We detected significant changes in the expression of genes encoding immunoproteasome catalytic subunits *Psmb8*, *Psmb9*, and *Psmb10* in astrocytes subjected to IFNγ treatment. Additionally, upregulation of the transcription of these genes was verified using real-time PCR (Figure 3b). Importantly, we also demonstrated the upregulation of mRNA related to immunoproteasome-associated REGα subunits, encoded by the *PSME1* gene [26], which is directly involved in antigen processing [27]. Our preliminary data suggest that REGα may enhance the entrapment of MBP by proteasomes [28], which—together with our previous reports [22,23,24]—suggest the elevated capability of IFNγ-treated astrocytes to process autoantigens, and MBP in particular.

## 3. Materials and Methods

### 3.1. Astrocytes Culture

The brains of 3-day-old C3H/He mouse pups were used as a source of astrocytes. All handling of animals was carried out in compliance with the protocols approved by the Commission of the Institute of Bioorganic Chemistry on the care and use of laboratory animals. The primary culture of astrocytes was obtained as described in Reference [29]. Briefly, a mixed astroglial–oligodendroglial cell culture was cultured for 8 days. Further, cells were washed using medium and were then separated by rotary shaking for 12 h at 250 rpm. Suspended cells containing oligodendrocytes were removed, and adhesion cells were washed using medium. Cells were detached with EDTA and were further plated at 3 × 10^4^/cm^2^. The culture medium was Basal Medium Eagle’s with Earle’s balanced salts containing 15% fetal calf serum, 0.1% glutamine, and 0.6% glucose. Cells, in independent triplicates, were treated using recombinant murine IFNγ (Sigma, I4777, St. Louis, MO, USA) at a concentration of 250 IU/mL [30], or with benztropine at a concentration of 2.0 μM [4], in culture medium for 48 h prior to analyses. Benztropin was kindly provided by Dr. Igor Titanyuk from the Department of Chemistry of M.V. Lomonosov Moscow State University.

### 3.2. Microarray Analyses

mRNA isolation and quality controls were performed as described in References [31,32]. The mRNA samples isolated from the three independent cell culture replicates were mixed in equal mRNA quantities. The procedures for cDNA synthesis and labelling were carried out according to the Ambion WT Expression Kit (Life Technologies, Darmstadt, Germany) using 500 ng of total RNA as a starting material, as described in Reference [33]. Target DNA fragmentation, hybridization on Affymetrix GeneChip Mouse Gene 2.0 ST microarrays, array washing, staining, and scanning were performed as described in Reference [34]. Scans of the microarrays were converted into CEL files using the scanner software, and were then processed in Affymetrix Expression Console (build 1.4.1.46) using the RMA method. Fold change threshold was set to 1.5×. Probesets with no associated Gene Symbol were excluded from the analyses. The raw data have been deposited to the Gene Expression Omnibus [35] under accession code GSE96899.

### 3.3. qPCR Validation of Gene Expression Data

RNA was reverse transcribed to cDNA as described in Reference [31] using 100 ng of RNA as a starting material. Quantitative PCR analysis was carried out using the SYBR Green 2.5× PCR reaction mix for qPCR (Syntol, Moscow, Russia). Primer pairs were designed and characterized as described in Reference [31]. PCR efficiencies of all primer sets were higher than 1.93 and lower than 2.06 (Supplementary Table S1), except for *H2-K1* (1.81). All RNA samples were analyzed in triplicate and averaged. Target genes were normalized to the reference genes *Tpt1*, *Ap1g1*, and *Eef1a1*, and data were processed based on the ΔΔCt method. Reference gene selection and validation were performed using the approach described in Reference [31].

## 4. Conclusions

Herein, we showed that exposure of a primary culture of murine cerebral cells significantly enriched in astrocytes to IFNγ significantly increases the expression of genes related to MHC class I/II molecules and immunoproteasome, and significantly enhances their capabilities of presenting exogenous and self-antigens. Evidently, the observed changes in the levels of mRNAs encoding MHC class I/II and immunoproteasome catalytic subunits may have a dualistic role—either enhancing the development of autoimmune neurodegeneration [36] or in the control of CNS infections [37]. IFNγ is known to induce the expression of catalytic immunosubunits of proteasomes [38] via intracellular signaling by STAT1 and IRF1 [39,40]. On the other hand, available data suggest that IFNγ-driven JAK-STAT-dependent induction of MHC class I and class II expression involves NOD-, LRR-, and CARD-containing 5 (NLRC5)—a specific transactivator of MHC class I genes (CITA) [41] and class II transactivator (CIITA) [42], respectively. In line with this, analyses of the expression of transcription factors in astrocyte cultures under IFNγ exposure (Figure 3c) revealed that these effects may be caused by JAK-STAT signaling, involving interferon regulatory factor 1 (IRF1), NLRC5 [43], and CIITA [44] (Figure 3d). In conclusion, our findings support the further study of astrocytes as antigen-presenting cells [45], and as targets for autoreactive cytotoxic lymphocytes [46].

## Supplementary Materials

The following are available online, Table S1: List of qPCR primers, Supplementary data: Cellular pathways affected by IFNg and benztropine.
