# Supplementary material for: The Transcriptome of Type I Murine Astrocytes under Interferon-Gamma Exposure and Remyelination Stimulus
Source: Molecules. 2017 May 15;22(5):808. doi: 10.3390/molecules22050808 (PMC6153759; doi:10.3390/molecules22050808)
Supplement: Supplementary file 1 [file molecules-22-00808-s001.zip › Supplementary_table_S1.docx]

| **Gene symbol** | **Primers sequences** | **qPCR primer efficiencies (E)** |
| --- | --- | --- |
| *Psmb6* | F-CTTTACGACGGTTGCTTTGTGG  R-ATTGGCGATGTAGGACCCAGT | 2.06±0.13 |
| *Psmb9* | F- TCTTCTGTGCCCTCTCAGGTTC  R- GCGCTAACAAGTCCTCACGGTA | 2.02±0.09 |
| *Psmb7* | F- ATGGAGGAGGAAGAAGCCAAGA  R- GGCACTGAGAATGGACGAAGAA | 1.95±0.08 |
| *Psmb10* | F- CGAGAACTGCCAGAGGAATG  R- ATTTTAGGGGCGATGAAGTGG | 1.93±0.09 |
| *Psmb5* | F- TCTGTGGCTGGGATAAGAGAGG  R- TCTGTAGGTGGCTTGGTAGATGG | 2.06±0.13 |
| *Psmb8* | F- AAGTGGTCATGGCGTTACTGGA  R- AACTTGAAGGCGAGTGTGGTTG | 1.94±0.09 |
| *Psmd1* | F- TGTCAGAAGAGCAGCAGTGGAG  R- CGTAGTTCACAGGGTCGTTGGT | 1.98±0.14 |
| *H-2K-sm1* | F- GCCCGCAGAACTCAGAAGTCGC  R- CCTGAATAGTGTGAGAGCCGCCC | 1.81±0.09 |
| *Eef1a1* | F- CAGTCGCCTTGGACGTTCTTT  R- TGCTTTGAATTAGCGGTGGTTT | 1.97±0.10 |
| *Tpt1* | F- TTGAAGAGCAGAAACCAGAAAGAGT  R- CCAGGAGAGCAACCATACCATC | 1.96±0.07 |
| *Ap1g1* | F- ACAGGGACCATCACACAAGTCA  R- GCCAGCAATCACAACCTCCTT | 1.97±0.12 |
